# Supplementary material for: Platelet-Derived PDGFB Promotes Recruitment of Cancer-Associated Fibroblasts, Deposition of Extracellular Matrix and Tgfβ Signaling in the Tumor Microenvironment
Source: Cancers (Basel). 2022 Apr 12;14(8):1947. doi: 10.3390/cancers14081947 (PMC9024906; doi:10.3390/cancers14081947)
Supplement: Supplementary file 1 [file cancers-14-01947-s001.zip › cancers-1672843-supplementary.pdf]

Supplementary Material: Platelet-Derived PDGFB Promotes Recruitment of Cancer-Associated Fibroblasts, Deposition of Extracellular Matrix and Tgfβ Signaling in the Tumor Microenvironment

Yanyu Zhang, Ehsan Manouchehri Doulabi, Melanie Herre, Jessica Cedervall, Qi Qiao, Zuoxiu Miao, Anahita Hamidi, Lars Hellman, Masood Kamali-Moghaddam and Anna-Karin Olsson

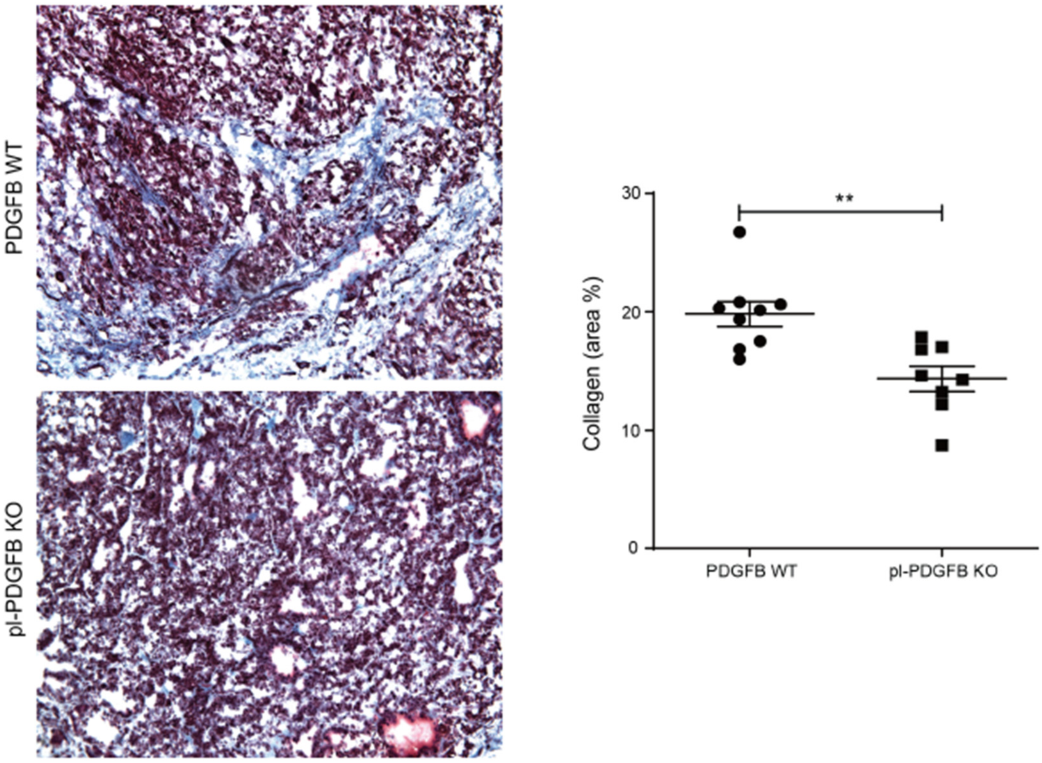

Figure S1. Reduced Col1 deposition in tumors from mice lacking PDGFB in platelets. Sections of tumor tissue from 14-weeks old WT and pl-PDGFB KO RT2- positive mice were stained with Masson trichrome for Col1 and the blue stained areas were quantified using Image J (WT n=9; KO n=8, *P*=0.0025). Error bars in the graphs represent the standard error of mean (SEM). ***p*=0.0025.

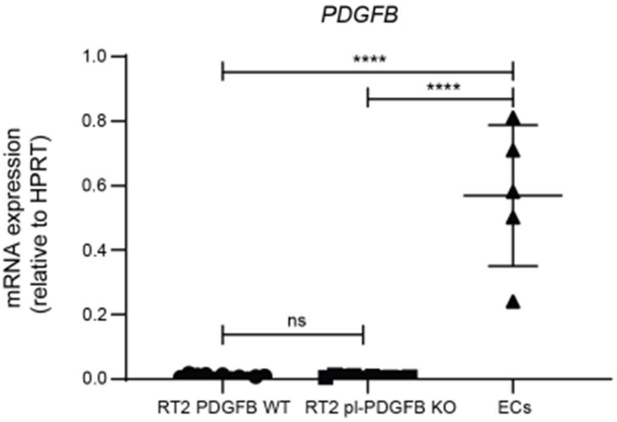

Figure S2. Endothelial cells are the major source of PDGFB transcription in RT2 tumors. Expression of PDGFB was analyzed by qPCR using RNA extracted from whole tumors or from endothelial cells (ECs) isolated from 14-weeks old WT and pl-PDGFB KO RT2-positive mice (WT n=11; KO n=10, ECs n=4, *P*=0.0005, 0.0007 and 0.7045 respectively). Error bars in the graphs represent the standard error of mean (SEM). ****p=0.0005 and 0.0007.

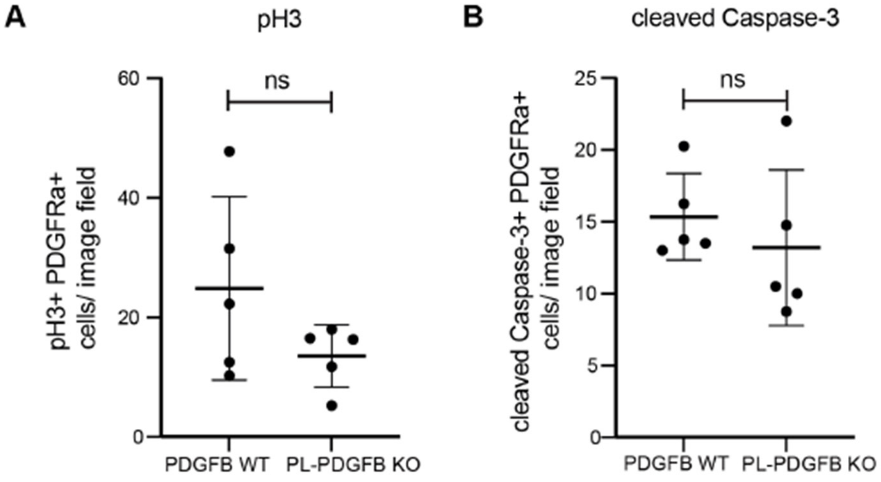

Figure S3. Proliferation and apoptosis of PDGFRα+ CAFs in tumor tissue from WT and pl-PDGFB KO mice. Tumor sections from 14-weeks old WT and pl-PDGFB KO RT2-positive mice were immunostained for PDGFRa (n=5/group) and coimmunostained for (A) phospho-histone 3 (pH3) to assess proliferation (*P*=0.3095) or (B) cleaved caspase-3 to assess apoptosis (*P*=0.4206). The positive area was quantified using Image J. Error bars in the graphs represent the standard error of mean (SEM).

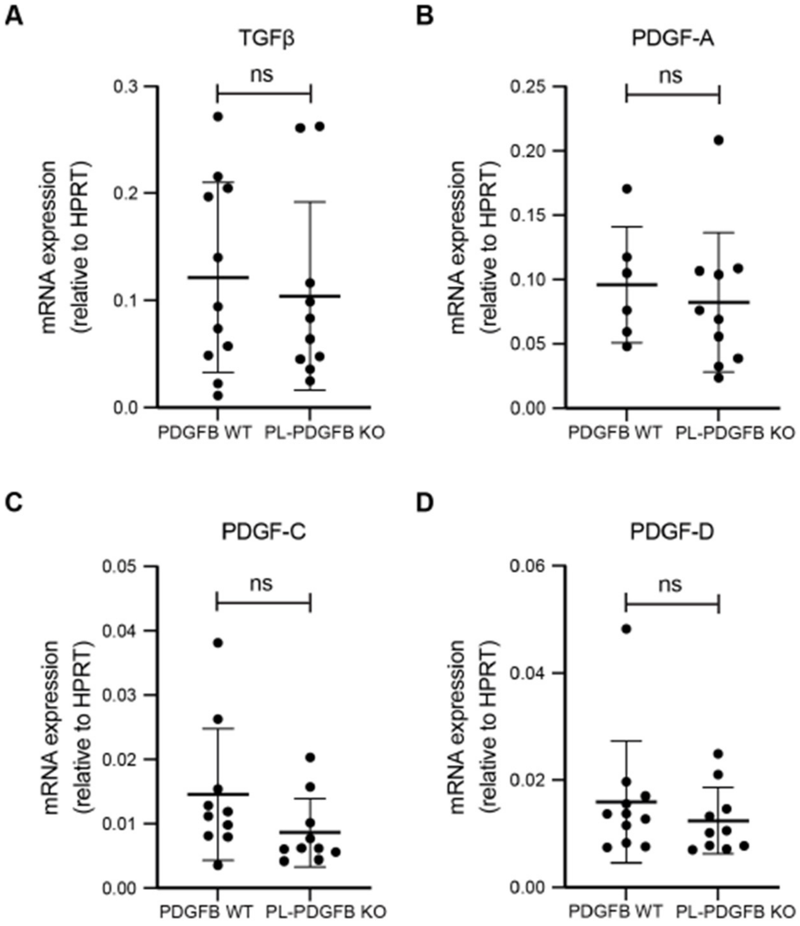

Figure S4. Expression levels of TGFb, PDGF-A, -C and -D are similar in tumor tissue from WT and pl-PDGFB KO mice. Expression of (A) TGFb (WT n=11; KO n=10, *P*=0.7045), (B) PDGF-A (WT n=6; KO n=10, *P*=0.4923), (C) PDGF-C (WT n=10; KO n=10, *P*=0.0892) and (D) PDGF-D (WT n=11; KO n=10, *P*=0.3867) was analyzed by qPCR using RNA extracted from whole tumors from 14-weeks old WT and pl-PDGFB KO RT2- positive mice. Error bars in the graphs represent the standard error of mean (SEM).

Publisher's Note: MDPI stays neutral with regard to jurisdictional claims in published maps and institutional affiliations.

© 2022 by the author. Licensee MDPI, Basel, Switzerland. This article is an open access article distributed under the terms and conditions of the Creative Commons Attribution (CC BY) license (https://creativecommons.org/licenses/by/4.0/).
